# Association of urinary uromodulin with kidney function decline and mortality: the health ABC study 

**DOI:** 10.5414/CN109005

**Published:** 2017-03-23

**Authors:** Pranav S. Garimella, Ronit Katz, Joachim H. Ix, Linda F. Fried, Stephen B. Kritchevsky, Prasad Devarajan, Michael R. Bennett, Chirag R. Parikh, Michael G. Shlipak, Tamara B. Harris, Orlando M. Gutiérrez, Mark J. Sarnak

**Affiliations:** 1 University of California San Diego, San Diego, CA,; 2 University of Washington, Seattle, WA,; 3 VA Pittsburgh Healthcare System and University of Pittsburgh, Pittsburgh, PA,; 4 Wake Forest School of Medicine, Winston-Salem, NC,; 5 University of Cincinnati, Cincinnati, OH,; 6 Yale University School of Medicine, New Haven, CT,; 7 Universtiy of California, San Francisco, CA,; 8 National Institute on Aging, Bethesda, MD,; 9 University of Alabama at Birmingham, AL, and; 10 Tufts Medical Center, Boston, MA, USA

**Keywords:** uromodulin, Tamm-Horsfall protein, tubular function, kidney function, CKD, mortality

## Abstract

Background: Urine uromodulin (uUMOD) is a protein secreted by the kidney tubule. Recent studies have suggested that higher uUMOD may be associated with improved kidney and mortality outcomes. Methods: Using a case-cohort design, we evaluated the association between baseline uUMOD levels and ≥ 30% estimated glomerular filtration rate (eGFR) decline, incident chronic kidney disease (CKD), rapid kidney function decline, and mortality using standard and modified Cox proportional hazards regression. Results: The median value of uUMOD was 25.8 µg/mL, mean age of participants was 74 years, 48% were women, and 39% were black. Persons with higher uUMOD had lower prevalence of diabetes and coronary artery disease (CAD), and had lower systolic blood pressure. Persons with higher uUMOD also had higher eGFR, lower urinary albumin to creatinine ratio (ACR), and lower C-reactive protein (CRP). There was no association of uUMOD with > 30% eGFR decline. In comparison to those in the lowest quartile of uUMOD, those in the highest quartile had a significantly (53%) lower risk of incident CKD (CI 73%, 18%) and a 51% lower risk of rapid kidney function decline (CI 76%, 1%) after multivariable adjustment. Higher uUMOD was associated with lower risk of mortality in demographic adjusted models, but not after multivariable adjustment. Conclusion: Higher levels of uUMOD are associated with lower risk of incident CKD and rapid kidney function decline. Additional studies are needed in the general population and in persons with advanced CKD to confirm these findings.

## Introduction 

The current assessment of kidney health and the definition of chronic kidney disease (CKD) are limited to measures or estimates of glomerular filtration rate (eGFR) and urinary albumin-creatinine ratio (ACR). Tubular health is essential for maintenance of acid-base status, mineral metabolism, and hormone production [1, 2]. In addition, tubular secretion is also the means by which medications are excreted through the kidneys. The kidney tubules may also have a role in preventing urinary infections. However, there are no biomarkers that have become accepted in clinical medicine as validated measures of tubular health. 

Urinary uromodulin (uUMOD), also known as Tamm-Horsfall protein, is a 95-kDa glycoprotein synthesized by the thick ascending limb of the loop of Henle and early distal convoluted tubule. It is the most abundant urinary protein among healthy adults (20 – 70 mg/day) [3]. Mutations in the UMOD gene are associated with low uUMOD levels and cause congenital hyperuricemic, cystic kidney diseases, and kidney failure [4, 5]. In large genome-wide association studies (GWAS), the strongest associations with CKD were with common variants in the region of UMOD gene [6, 7]. As a result, there has been a renewed interest in the role of uUMOD in the development and progression of kidney disease. 

Recently, our group has found that among participants enrolled in Cardiovascular Health Study (CHS), each standard deviation (SD) higher uUMOD was associated with 23% lower odds of eGFR decline in models adjusted for demographics, eGFR, ACR, and CKD risk factors [8]. In addition, each SD higher uUMOD was also associated with 10% lower risk of mortality in adjusted models. 

In the Health, Aging & Body Composition study (Health ABC), we aimed to validate our previous work and evaluate the association between uUMOD levels, kidney function decline, and mortality in older adults. We hypothesize that lower uUMOD concentrations may serve as a noninvasive method of assessing impaired tubular health and identifying persons at higher risk for progressive kidney disease and mortality. 

## Methods 

### Participants 

The Health ABC is a longitudinal study of the impact of changes in weight and body composition on age-related physiologic and functional changes. Community-dwelling adults (n = 3,075) aged 70 – 79 years were recruited from Medicare eligibility lists from March 1997 through July 1998 at two field centers in Pittsburgh, PA, and Memphis, TN. White participants were recruited from a random sample of the lists; black participants were recruited from all age-eligible individuals residing in the respective communities. Subjects were eligible if they reported no difficulty walking one-fourth of a mile, climbing up 10 steps, or performing basic activities of daily living; were free of life-threatening illness; planned to remain in the geographic area for ≥ 3 years; and were not enrolled in lifestyle intervention trials. Exclusion criteria included difficulties with activities of daily living, cognitive impairment, inability to communicate with the interviewer, intention of moving within the next 3 years, and active treatment for cancer in the preceding 3 years. All participants gave informed consent, and the study was approved by the institutional review boards at the University of Tennessee Health Science Center and the University of Pittsburgh. 

### Study design 

**Figure 1.**
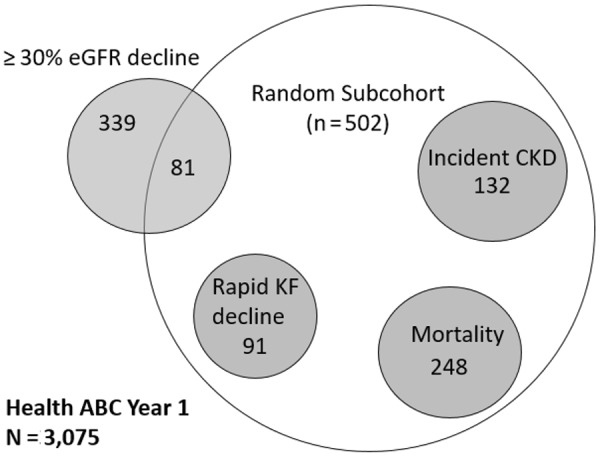
Selection of study participants. Sampling strategy for the present study. Of 3,075 participants at year 1, a representative random subcohort of 502 participants was created. All cases of ≥ 30% eGFR decline from outside the subcohort were sampled to maximize statistical power. Cases of mortality and the secondary kidney outcomes of rapid kidney function decline and incident CKD were from within the subcohort. KF = kidney function.

We used a case-cohort design for this study, and Figure 1 depicts the sampling design for this study strategy. From the entire cohort of Health ABC (n = 3,075), we selected a random subcohort of 502 participants at year 1. Our primary outcomes were ≥ 30% eGFR decline and mortality. In addition to the random subcohort, cases were selected for ≥ 30% eGFR decline to ensure sufficient number of events and adequate statistical power. We defined decline as ≥ 30% decline in eGFR from the year-1 visit to the year-10 visit. If year 10 was missing, we used eGFR from the year-3 visit. Overall, there were 420 participants who had ≥ 30% decline in eGFR during the study period. Of these, 81 were already in the random subcohort, and an additional 339 participants with > 30% eGFR decline were identified from the entire Health ABC study population. As secondary outcomes, we also assessed for incident CKD (eGFR < 60 mL/min/1.73m^2^ and at least 1 mL/min/1.73m^2^/year decline in eGFR in individuals with eGFR ≥ 60 mL/min/1.73m^2^ at baseline) and rapid kidney function decline (> 3 mL/min/year decline in eGFR) in the subcohort only. As there were sufficient mortality events (n = 248) from within the random subcohort, we did not additionally sample for mortality. In aggregate, the random subcohort and the CKD progression cases provided an analysis cohort of 841 participants. 

### Exposure variable 

Spot urine specimens were obtained at the time of the year-1 study visit and stored at –70 °C until thaw and uUMOD was measured at the University of Cincinnati Children’s Hospital Medical Center in 2015. Uromodulin was assayed in the urine using a commercially-available ELISA kit (MD Bioproducts, St. Paul, MN, USA) according to the manufacturer’s instructions. The assay is based on a colorimetric sandwich immunoassay utilizing a polyclonal antibody against human uromodulin as the capture antibody and a biotinylated polyclonal antibody against human uromodulin as the detection antibody. The correlation coefficient for this particular assay run was 1.00. The interassay coefficient of variation obtained by analyzing results from 81 duplicate measures spread between 10 separate assay runs was as 5.02%. The minimum detectable concentration as reported by the manufacturer was 0.75 ng/mL. No measured values in our cohort were below the detectable limit. 

### Outcomes 

The eGFR was estimated using an equation that included serum cystatin C concentration, age, sex, and race derived in the CKD-EPI study [9]. A 30% decline in eGFR has recently been associated with adverse outcomes and therefore recommended as an alternative endpoint for CKD progression [10]. In secondary analysis, we used incident CKD (eGFR < 60 at year 3 or 10 and at least 1 mL/min decline in eGFR) and rapid kidney function decline defined by > 3 mL/min/year decline in eGFR, given their association with adverse clinical outcomes in prior studies [11]. Cystatin C was measured using a BNII nephelometer (Dade Behring Inc., Deerfield, IL, USA) that used a particle-enhanced immunonephelometric assay (N Latex Cystatin C) [12]. We used cystatin C-based eGFR estimates as creatinine was not isotope dilution mass spectrometry (IDMS) standardized at all the visits during follow-up thus increasing the risk of bias due to measurement variability. 

The Health ABC Study Diagnosis and Disease Ascertainment Committee reviewed all hospital records, death certificates, informant interviews, and autopsy data to adjudicate immediate and underlying causes of death. 

### Covariates 

Covariates for the models were selected a priori based on biological plausibility and prior published data regarding potential confounders of the associations of uUMOD with the clinical endpoints [8]. A series of sequential models were fit to evaluate the effects of adding certain sets of covariates. Traditional cardio-vascular disease (CVD) and kidney disease risk factors including age, gender, race/ethnicity, body mass index (BMI), physical activity level, smoking defined by current versus former (> 100 lifetime cigarettes) or never, diabetes (defined by use of hypoglycemic agents, fasting plasma glucose > 126 mg/dL or nonfasting glucose ≥ 200 mg/dL), systolic blood pressure, low-density lipoprotein cholesterol level (LDL), high-density lipoprotein cholesterol level (HDL), statin medications, blood pressure medications including angiotensin-converting enzyme inhibitors, angiotensin receptor blockers, calcium channel blockers, diuretics, and C-reactive protein (CRP) were considered as confounding variables. Analyses were also adjusted for baseline eGFR and ACR. 

### Statistical analysis 

We described the distribution of uUMOD using summary statistics and frequency histograms and compared baseline participant characteristics (demographics, kidney function, CVD risk factors) by uUMOD quartiles among members of the subcohort. We evaluated the correlations of uUMOD, eGFR, and albuminuria using Spearman correlation coefficients. We evaluated each uUMOD-outcome association for the presence of nonlinearity using generalized additive models and restricted cubic splines. Risk estimates were calculated by categories of uUMOD (defined by quartiles of the distribution among the random subcohort) for all outcomes. 

We evaluated the association of uUMOD with eGFR decline, using modified Cox regression to account for the case-cohort approach [13, 14]. Subcohort participants were weighted with the inverse of the sampling fraction. Cases that arose outside the subcohort were not weighted before their failure. All cases (irrespective of whether they arose in the subcohort or not) were assigned a weight of 1 at the time of failure. For each outcome, we fit a series of sequential models. Initial models were adjusted for age, sex, race, and clinic site. A subsequent model additionally adjusted for baseline eGFR and urine ACR. A final model included CVD and CKD risk factors (body mass index, diabetes, systolic blood pressure, blood pressure medication use, total cholesterol, lipid-lowering medication, CRP, and smoking status). We evaluated associations of uUMOD with mortality using a standard Cox proportional hazards regression. For the secondary analysis of kidney outcomes (incident CKD and rapid kidney function decline), we used standard Cox proportional hazards regression as these cases were restricted to the random subcohort, and no additional sampling was performed. The proportional hazards assumption was tested based on an extended version of the Schoenfeld residuals weighted Cox models [15]. For each of the clinical outcomes, we evaluated whether the uUMOD-outcome association was modified by CKD status (defined as eGFR < 60 mL/min/1.73m^2^). 

Analyses were conducted using R version 3.2.2 (2015-08-14) (R Core Team, Vienna, Austria) and IBM SPSS Statistics for Windows, Version 23.0. (IBM Corp, Armonk, NY, USA) [16]. Estimates with two-sided p-values < 0.05 were considered statistically significant. 

## Results 

### Baseline characteristics 

Among the 502 randomly-selected participants at baseline, the median (Q1, Q3) value of uUMOD was 25.8 µg/mL (15.8, 40.2), and the distribution was right skewed. The mean ± SD age of participants was 74 ± 3 years, 48% were women, and 39% were black. The mean ± SD eGFR was 73 ± 18 mL/min/1.73 m^2^, and median (Q1, Q3) urine ACR was 5 (4, 20) mg/g. Table 1 shows baseline characteristics by quartiles of uUMOD in the random subcohort. Compared to participants with low uUMOD, persons with higher levels were less likely to have diabetes or history of coronary artery disease (CAD), and had lower systolic blood presuure (BP). Persons with higher uUMOD also had higher eGFR, lower urinary ACR, and lower CRP. At baseline, uUMOD levels were weakly and inversely correlated with ACR (Spearman r = –0.11). 

### uUMOD and kidney outcomes 

There were 420 participants with ≥ 30% decline of eGFR during the follow-up period. There was no association of uUMOD when analyzed as a continuous variable (per SD increase) or as quartiles with ≥ 30% eGFR decline (Table 2). In secondary analyses restricted to only the random subcohort, there were 91 incident CKD events and 132 participants who experienced rapid kidney function decline. Compared to the participants in the first quartile, participants in the fourth quartile of uUMOD had > 50% lower hazard of incident CKD (HR 0.47 (0.27, 0.82)), after adjusting for confounders. Similarly, persons in the highest quartile of uUMOD also had a statistically significant lower hazard of rapid kidney function decline compared to persons in the lowest quartile of uUMOD (HR 0.49 (0.24, 0.99)). 

### uUMOD and mortality 

There were 248 deaths within the subcohort over the duration of follow-up. After adjusting for age and demographics, each 1-SD higher uUMOD was associated with a 16% lower risk of death. This association lost statistical significance after adjusting for additional comorbidities and eGFR. After multivariable adjustment, the highest quartile of uUMOD was not associated with a statistically-significant risk of death in comparison with the lowest quartile (HR 0.79, (0.53, 1.17)) (Table 2). 

**Figure 2. Figure2:**
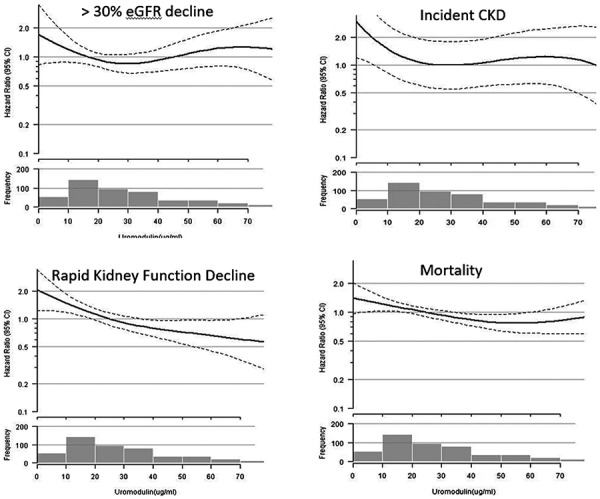
Unadjusted restricted cubic splines evaluating the association between uUMOD and outcomes. Left to right and top to bottom: > 30 glomerular filtration rate (GFR) decline, incident chronic kidney disease (CKD), rapid kidney function decline (> 3 mL/min/year), and mortality. Each model was fitted using a restricted cubic spline function for urinary uromodulin (uUMOD). In each plot, the solid line represents the curve estimates, and the dotted lines represent 95% confidence bands. The histogram below the cubic splines represents the frequency of events per 10 µg/mL uUMOD increments.

We found no evidence of interaction between eGFR < 60 and uUMOD with the outcomes of ≥ 30% eGFR decline (p = 0.33), incident CKD (p = 0.14) or rapid kidney function decline (p = 0.80), and mortality (0.98). [Fig Figure2]

## Discussion 

In summary, for two of our three kidney outcomes, and for mortality, we noted that higher levels of uUMOD were associated with a lower risk, although the results were not statistically significant in all models. These findings are consistent, in direction and magnitude, with prior data in older adults wherein we demonstrated that persons in the highest quartile of uUMOD had a lower risk of ≥ 30% eGFR decline and mortality [8]. 

The mechanisms linking uUMOD to kidney function decline may involve the prevention of urinary infections [17], stone formation [18] and its anti-inflammatory effects after tubular injury [19], although a clear causal role has yet to be established. As uUMOD is produced by kidney tubule cells, it is also possible that higher levels of uUMOD indicate better tubular function, such as erythropoietin production, maintenance of acid-base and mineral metabolism homeostasis. 

Prior studies evaluating the association of uUMOD with kidney disease have shown conflicting results [6, 8, 20, 21]. These may in part be explained by select patient populations, small sample sizes, severity of underlying kidney disease, and differences in uUMOD assays. It has also been hypothesized that during renal injury, uUMOD may be upregulated and contribute to the repair of the kidney, whereas persistent injury and inflammation may result in fixed or lower levels of uUMOD [22], thus resulting in a bimodal association as has been demonstrated previously [23]. It has been postulated that polymorphisms in the UMOD gene seen in GWAS studies [6, 24] may lead to a phenotype with increased uUMOD expression, decreased membrane-anchoring efficiency, and faster protein trafficking or increased proteolytic release, which is associated with an increased risk of adverse clinical events [25]. 

Recently, two studies evaluated the clinical correlates of uUMOD in large population cohorts. The first study included two cohorts; the Swiss Kidney Project on Genes in Hypertension study in which 817 participants with kidney ultrasound measures provided 24-hour urine collections, and the Cohorte Lausannoise study cohort in which 5706 adults provided morning spot urine samples [26]. In the second study, data from 943 participants in the CARTaGENE study were analyzed to study the determinants of uromodulin excretion in the general population aged 40 – 69 years [27]. Both studies reported significant positive correlation between uUMOD levels and eGFR after adjusting for multiple confounders. In addition, a positive correlation was found between higher uUMOD excretion and urinary sodium, chloride, and uric acid excretion, suggesting a potential role in salt and water homeostasis. Further, a positive linear correlation between uUMOD and kidney length and volume led the investigators to hypothesize that uUMOD may be a surrogate for kidney tubular function and reserve [26]. Additionally, two more recent studies in humans have shown that higher uUMOD levels are associated with a significantly lower risk of urinary tract infection (UTI) in community-dwelling older adults [17] and with lower risk of AKI in persons undergoing cardiac surgery [28]. Our study adds to this growing body of literature suggesting that higher uUMOD levels may be associated with better kidney function and clinical outcomes. 

Our study is limited by a number of factors that should be considered. First, participants in the Health ABC cohort, although elderly, had relatively well-preserved kidney function. It is therefore unknown whether uUMOD would be differentially associated with outcomes in those with proteinuria and more advanced kidney disease. Although we found no evidence for an interaction with CKD, the small sample size limits the statistical power. Second, we used a single measure of kidney function at follow-up to determine eGFR decline, incident CKD, and rapid kidney function decline, which should ideally have been confirmed by a second value. Third, the findings of higher uUMOD being associated with less kidney function loss was limited to our secondary outcomes of incident CKD and rapid kidney function decline, and therefore need to be interpreted cautiously. Fourth, the samples in our study had been stored for nearly 15 years prior to analysis. Although storage even at –80 °C for 8 months can decrease levels of uUMOD [29], we believe this should be nondifferential with respect to the clinical outcomes. Our study also has a number of strengths. The Health ABC cohort has detailed ascertainment of risk factors, covariates, and outcomes as well as the ability to adjust for measures of kidney function. We used a laboratory that has extensive experience with these measures including using the same assay as has been done in prior studies [8, 28]. 

In summary, results from our study are consistent with prior data indicating that higher levels of uUMOD may imply “healthier tubules” and better prognosis from the kidney and mortality standpoint. These findings improve our understanding of the different facets of kidney health and function above and beyond the traditional measures of eGFR and ACR. Studies are needed in more generalizable populations and those with more advanced stages of CKD to evaluate whether higher levels of uUMOD are similarly associated with better kidney and mortality outcomes. 

## Funding 

This research was supported in part by the Intramural Research Program of the National Institutes of Health (NIH) and the National Institute on Aging (NIA) Contracts N01-AG-6-2101; N01-AG-6-2103; N01-AG-6-2106; NIA grant R01-AG028050, and NINR grant R01-NR012459. Drs Shlipak, Sarnak, Ix, and Katz were supported by NIA grant 5R01AG027002, and Dr. Ix by an American Heart Association Award #14EIA18560026 and NIDDK:K24DK110427. Dr. Devarajan was supported by National Institutes of Health (NIH) grant P50 DK096418. The study sponsors had no role in study design; collection, analysis, and interpretation of the data; writing the report; and the decision to submit the report for publication. 

**Table 1.** Baseline characteristics of older community-living adults by quartiles of urine uromodulin (uUMOD).

uUMOD, µg/mL					
	Quartile 1	Quartile 2	Quartile 3	Quartile 4	All
	≤ 15.80	15.81 – 25.86	25.87 – 40.27	> 40.27	
N	126	125	126	125	502
Age	74 ± 3	74 ± 3	74 ± 3	73 ± 3	74 ± 3
Female	48	54	47	46	48
Black	41	39	33	43	39
Site					
Memphis	54	58	48	48	52
Pittsburgh	46	42	52	52	48
Education					
< High school	26	24	20	22	23
High school	41	31	38	26	34
Postsecondary	33	45	42	52	43
Diabetes	33	21	23	20	24
Hypertension	70	65	60	63	65
SBP	138 ± 22	134 ± 22	132 ± 19	133 ± 20	134 ± 21
Smoking					
Never	41	42	40	38	40
Former	48	53	52	54	52
Current	10	5	8	8	8
BMI, kg/m^2^	27 ± 4	27 ± 4	27 ± 5	27 ± 4	27 ± 4
CRP*, mg/L	1.81 [1.07, 3.70]	1.50 [0.95, 3.27]	1.47 [1.00, 2.72]	1.64 [0.91, 2.70]	1.63 [0.98, 2.95]
LDL cholesterol, mg/dL	118 ± 35	118 ± 35	122 ± 34	123 ± 36	121 ± 35
HDL cholesterol, mg/dL	52 ± 17	56 ± 18	52 ± 17	52 ± 16	53 ± 17
eGFR, mL/min/1.73m^2^	70 ± 21	74 ± 18	74 ± 17	74 ± 17	73 ± 18
ACR*, mg/g	12 [5, 37]	8 [5, 17]	6 [4, 15]	8 [4, 17]	5 [4, 20]
Calcium (n = 474)	8.8 ± 0.4	8.8 ±0.4	8.8 ± 0.4	8.8 ± 0.4	8.8 ± 0.4
Phosphorus (n = 470)	3.6 ± 0.5	3.6 ± 0.4	3.5 ± 0.5	3.5 ± 0.5	3.5 ± 0.5
PTH (n = 475)	34 [26, 47]	31 [23, 44]	31 [24, 41]	34 [25, 43]	32 [25, 44]

SBP = systolic blood pressure; BMI = body mass index; CRP = C-reactive protein; LDL = low-density lipoprotein, HDL = high-density lipoprotein; eGFR = estimated glomerular filtration rate; ACR = albumin to creatinine ratio; PTH = parathyroid hormone. All values represented as % or mean ± SD except those marked with * indicating median [interquartile range].

**Table 2.** Association of uUMOD with kidney outcomes and mortality in older community-living adults.

	uUMOD quartiles				Linear model	
	1	2	3	4	Per SD increase = 27.1 µg/mL	p-value
	≤ 15.80	15.81 – 25.86	25.87 – 40.27	> 40.27		
≥ 30 eGFR decline (case-cohort design)						
N	126	125	126	124	501	
Events	26	10	23	22	81	
Incidence rate (%/yr)*	3.22	1.2	2.38	2.49	2.32	
Model 1**	1.00 (ref)	0.74 (0.48, 1.13)	0.64 (0.41, 0.99)	0.90 (0.58, 1.40)	1.02 (0.86, 1.20)	0.864
Model 2^†^	1.00 (ref)	0.80 (0.52, 1.23)	0.78 (0.50, 1.21)	0.94 (0.60, 1.49)	1.03 (0.86, 1.23)	0.792
Model 3^‡^	1.00 (ref)	0.82 (0.53, 1.26)	0.93 (0.60, 1.44)	1.10 (0.69, 1.75)	1.08 (0.90, 1.29)	0.418
Incident CKD (subcohort only)						
N	126	125	126	124	501	
Events	46	31	33	22	132	
Incidence rate (%/yr)	5.70	3.72	3.41	2.49	3.78	
Model 1**	1.00 (ref)	0.62 (0.39, 0.99)	0.50 (0.31, 0.80)	0.40 (0.23, 0.69)	0.75 (0.58, 0.97)	0.029
Model 2^†^	1.00 (ref)	0.69 (0.43, 1.11)	0.59 (0.36, 0.97)	0.44 (0.25, 0.76)	0.77 (0.61, 0.99)	0.037
Model 3^‡^	1.00 (ref)	0.68 (0.41, 1.11)	0.63 (0.38, 1.05)	0.47 (0.27, 0.82)	0.79 (0.52, 1.01)	0.055
Rapid kidney function decline (subcohort only)						
N	88	103	104	94	389	
Events	28	15	32	16	91	
Incidence rate (%/yr)	4.79	2.17	3.91	2.40	3.3	
Model 1**	1.00 (ref)	0.35 (0.17, 0.69)	0.67 (0.39, 1.16)	0.43 (0.22, 0.83)	0.73 (0.52, 1.02)	0.064
Model 2^†^	1.00 (ref)	0.37 (0.18, 0.75)	0.66 (0.38, 1.16)	0.50 (0.25, 0.98)	0.77 (0.55, 1.07)	0.123
Model 3^‡^	1.00 (ref)	0.35 (0.16, 0.73)	0.60 (0.33, 1.10)	0.49 (0.24, 0.99)	0.76 (0.55, 1.06)	0.109
All-cause mortality (subcohort only)						
N	126	125	126	125	502	
Events	68	68	55	57	248	
Incidence rate (%/yr)	5.40	5.23	3.85	4.28	4.66	
Model 1**	1.00 (ref)	0.87 (0.61, 1.24)	0.57 (0.39, 0.83)	0.63 (0.43, 0.92)	0.81 (0.68, 0.96)	0.017
Model 2^†^	1.00 (ref)	0.95 (0.67, 1.36)	0.65 (0.44, 0.94)	0.70 (0.48, 1.02)	0.84 (0.71, 0.99)	0.040
Model 3^‡^	1.00 (ref)	1.09 (0.75, 1.59)	0.74 (0.50, 1.10)	0.79 (0.53, 1.17)	0.86 (0.73, 1.02)	0.084

*Incidence rate for > 30 eGFR decline is calculated from the random subcohort only. **Adjusted for age, gender, race, education, and site. ^†^Additionally adjusted for estimated glomerular filtration rate and urine albumin-creatinine ratio. ^‡^Additionally adjusted for diabetes, systolic blood pressure, antihypertensive medications, smoking status, body mass index, low-density lipoprotein cholesterol, high-density lipoprotein cholesterol, statin use, and C-reactive protein.
